# Intercorrelation Limits in Molecular Descriptor Preselection for QSAR/QSPR

**DOI:** 10.1002/minf.201800154

**Published:** 2019-04-04

**Authors:** Anita Rácz, Dávid Bajusz, Károly Héberger

**Affiliations:** ^1^ Plasma Chemistry Research Group Research Centre for Natural Sciences Hungarian Academy of Sciences Magyar tudósok krt. 2 1117 Budapest Hungary; ^2^ Medicinal Chemistry Research Group Research Centre for Natural Sciences Hungarian Academy of Sciences Magyar tudósok krt. 2 1117 Budapest Hungary

**Keywords:** analysis of variance, correlation, descriptor, QSAR, regression, sum of ranking differences

## Abstract

QSAR/QSPR (quantitative structure‐activity/property relationship) modeling has been a prevalent approach in various, overlapping sub‐fields of computational, medicinal and environmental chemistry for decades. The generation and selection of molecular descriptors is an essential part of this process. In typical QSAR workflows, the starting pool of molecular descriptors is rationalized based on filtering out descriptors which are (i) constant throughout the whole dataset, or (ii) very strongly correlated to another descriptor. While the former is fairly straightforward, the latter involves a level of subjectivity when deciding what exactly is considered to be a strong correlation. Despite that, most QSAR modeling studies do not report on this step. In this study, we examine in detail the effect of various possible descriptor intercorrelation limits on the resulting QSAR models. Statistical comparisons are carried out based on four case studies from contemporary QSAR literature, using a combined methodology based on sum of ranking differences (SRD) and analysis of variance (ANOVA).

## 1 Introduction

From the landmark works of Hammett in the 1930s,^1^ and Hansch and Fujita in the 1960s,^2^, ^3^ quantitative structure‐activity, and structure‐property relationships (QSAR/QSPR) have come a long way, evolving into a ubiquitous concept, present in many sub‐fields of chemistry. QSAR studies are the subjects of hundreds of publications yearly, and the state of the field is thoroughly summarized from time to time.^4^

The importance of QSAR was even recognized by the Organization for Economic Co‐operation and Development (OECD), who have published a set of principles (along with a detailed guidance document) for QSAR model validation.^5^ Also, in the last decades, researchers of this field have been concerned with identifying and promoting best practices for QSAR modelling and validation.^6^, ^7^, ^8^, ^9^

Descriptor (pre‐)selection is an integral part of QSAR workflows. It usually involves the removal of descriptors with missing values, constant values across the whole dataset, or collinear (inter‐correlated) descriptors. The entire process is called variable reduction according to terms of Todeschini and Consonni: “Variable reduction consists of the selection of a subset of variables able to preserve the essential information contained in the whole dataset but eliminating redundancy, too highly correlated variables, etc. Variable reduction differs from variable selection in the fact that the subset of variables is selected independently from the response of interest.”^10^ While the removal of constant (or nearly constant) variables is relatively straightforward, there is no clear consensus on the choice of the specific intercorrelation limit in QSAR studies: a random sample from the QSAR literature of the recent years reveals choices of 1.000,^6^ 0.98,^11^ 0.95,^12^, ^13^ 0.90,^14^, ^15^ 0.80,^16^ and even 0.70.^17^ Moreover, most studies either do not report the selected intercorrelation limit,^18^, ^19^ or simply omit this step.

It should be noted that some of the new‐generation variable selection and QSAR modeling methods (such as PLS regression or principal component regression, PCR) inherently neglect redundant variables (rendering variable reduction unnecessary for small datasets), however these methods are not necessarily implemented in the relevant and popular QSAR modeling software. Also, variable reduction is useful especially in the case of large datasets to save computational time even (and especially) for advanced regression methods, and similarly to variable selection tools such as genetic algorithms.

A relatively straightforward and popular method (the QUIK rule) was proposed in 2004 by Todeschini *et al*. for collinearity detection among the descriptors (it is even suggested by OECD in their guidance document).^20^ The QUIK rule is based on checking the total correlation of the set of descriptors in the QSAR model (*K_XX_*, based on the *K* multivariate correlation index) against that of the model descriptors plus the response variable (*K_XY_*) and rejecting any model where the difference of the two is not large enough. While this is a successful and popular method to check for collinearity *after* modelling, it does not provide guidance regarding the amount of descriptors to remove from the dataset (in order to improve the model), if the collinearity is found to be too large.

Our goal with this work is to propose guidelines for selecting the intercorrelation limit for descriptor selection in QSAR modelling. (Throughout the article, we use the term “intercorrelation” to refer to the correlation between two descriptors, rather than a descriptor and the modeled property.) We report a detailed statistical comparison of QSAR models generated with a wide range of intercorrelation limits, based on four case studies from the literature, with diverse endpoints. It is worth to stress that our purpose was to compare the intercorrelation limits fairly, and not to build the best models, which has already been done in the original publications.^23^, ^24^, ^25^, ^26^, ^27^ We hope to provide general conclusions using these highly diverse data sets. In any modeling task, there are many suboptimal solutions indistinguishable in the statistical sense. SRD as a multicriteria decision making tool is able to distinguish suboptimal solutions.^6^, ^21^ Following our recent comparative studies,^22^, ^23^ the combination of sum of ranking differences (SRD) and analysis of variance (ANOVA) is applied for the evaluations.

## 2 Materials and Methods

### 2.1 Datasets

Four case studies were used for the analysis. The first dataset (**Dataset 1**) contained IC_50_ values of novel N‐benzoyl‐L‐biphenylalanine derivatives as potent inhibitors for α4 integrins.^24^ The second (**Dataset 2**) was an ADME properties evaluation study, which contained the logBB values (blood‐brain partitioning coefficient) of more than three hundred compounds.^25^ The third (**Dataset 3**) was a toxicology study of benzene derivatives, where the toxicity values were expressed as acute toxicities (pLC_50_) for the fathead minnow (*Pimephales promelas*).^26^ In the fourth case study (**Dataset 4**), pIC_50_ values were reported for the hMGL enzyme (human monoglyceride lipase) for a set of N‐substituted maleimides.^27^ Specific details of the used datasets are summarized in Table 1. Smiles and SDF files of the datasets were used for molecular descriptor generation.^28^

**Table 1:** Number of compounds in the training and test sets with endpoints and references, for the four case studies.

	Endpoint	Applicability domain	No. training	No. test	Ref.
1	pIC_50_	N‐benzoyl‐L‐biphenylalanine derivatives	99	43	[24]
2	logBB	Diverse compounds	287	81	[25]
3	pLC_50_	Benzene derivatives	51	18	[26]
4	pIC_50_	N‐substituted maleimides	48	14	[19,27]

### 2.2 Molecular Descriptor Generation

**Figure 1:**
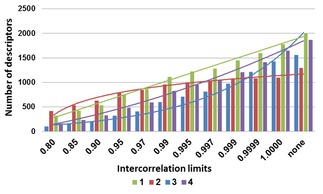
Numbers of selected descriptors for the four datasets, for each intercorrelation limit. (“none” means that no correlation limit was used.)

In total 3839 (2D) descriptors were generated with the DRAGON 7 software.^29^ Descriptors with constant values and descriptors with at least one missing value were excluded. Next, the absolute intercorrelation limit between descriptors was set to: 0.8000, 0.8500, 0.9000, 0.9500, 0.9700, 0.9900, 0.9950, 0.9970, 0.9990, 0.9999, 1.0000 or None (no limit). For each pair of correlated descriptors, the one showing the highest pair correlation with the other descriptors was automatically excluded.^30^ Every descriptor set, corresponding to different limits, was saved for the model building phase. The selected number of descriptors can be seen in Figure 1 for each dataset. This figure also highlights the dataset dependence of the number of descriptors selected by the different intercorrelation limits.

### 2.3 Model Building

In the next step, Gramatica and coworkers’ QSARINS 2.2.2 software (http://www.qsar.it) was used for model building.^31^, ^32^ This software has a rich toolbox of statistical methods for model generation, validation (internal and external) and it can output several performance parameters for the models, as well.^6^ To focus solely on the effect of the intercorrelation limits, we have used the same settings for all of the four case studies during model building.

The models were calculated by multiple linear regression (MLR) with ordinary least squares (OLS), and a genetic algorithm (GA) was used for variable selection.^33^ The *Q* ^2^ for leave‐one‐out cross‐validation (*Q* ^2^_LOO_) was applied as the objective function in GA, which is a standard and widely‐accepted choice for regression models.^34^, ^35^, ^36^ The number of populations was 100, and 100 iterations were performed. The mutation probability was set to 20 %, and the maximum number of variables included in the model was ten.

The datasets were split into training and test sets based on the original articles, and were kept fixed. For model selection, we suggest – in compliance with generally approved practices – using the same performance parameter that was used as the objective function during variable selection (here, *Q* ^2^_LOO_). However, in the present study, typically more than one model was produced (for each specific dataset and intercorrelation limit) with very similar *Q* ^2^_LOO_ values (having statistically insignificant differences only). From these, the final models were selected based on their *R* ^2^ values for the further evaluations.

### 2.4 Statistical Comparison

Sum of ranking differences (SRD) was used for the comparison of the selected models.^37^, ^38^ SRD is a novel, robust statistical method for model/method comparison, based on the use of an “ideal” reference method (gold standard, benchmark). The reference can be a set of experimentally determined values, but it is possible to use a hypothetical consensus method based on data fusion possibilities *(e. g*.: average, minimum, maximum, *etc*.), as well. By convention, the input matrix contains the variables (models, methods) in the columns and the samples (here, molecules) in the rows. The procedure is based on the following steps: (i) ranking the samples in order of magnitude according to each models and the reference, (ii) calculating the absolute rank differences for each molecule, between each model and the reference, and (iii) summing up the calculated differences for each model. The resulting sums are called SRD (sum of ranking differences) values (or SRD scores) and they represent the City block (Manhattan) distances of the models from the reference. (A smaller SRD value thus means proximity to the “ideal” reference, hence the smaller the better.) A detailed illustration about the SRD procedure can be found as a supplement to our earlier work.^39^ SRD values can be compared for different studies with the use of normalized SRD (*SRD_nor_*) values:

(1) $$\mathrm{SRD}{}_{\mathrm{nor}}=100\;\mathrm{SRD}/\mathrm{SRD}{}_{\max}$$

where *SRD_max_* means the theoretical maximum of SRD values.

Sum of ranking differences employs two types of validation. Comparison of ranks with random numbers (CRRN) is a randomization test, which gives a distribution of SRD values with randomized ranks. Based on this validation, one can conclude whether the SRD value characterizing a model overlaps with the use of random numbers (if so, then the model is statistically not distinguishable from randomly assigned ranks). *N*‐fold cross‐validation (or leave‐one‐out or leave‐many‐out cross‐validation) is applied to check whether the SRD values of two models (methods, *etc*.) are significantly different. Here, the contiguous (block‐wise) and a repeated sampling version of sevenfold cross‐validation was used. (This version corresponds to Monte Carlo sampling, which allows overlap between the training/test splits of the different iterations.)

In the present study, QSAR/QSPR models – calculated with descriptors that were selected with different intercorrelation limits – were compared. The input matrices contained endpoints (pIC_50_, logBB, *etc*.) predicted by the various QSAR models (columns) for each molecule of the datasets (rows), with the experimentally determined endpoints as the reference in each case. Two forms of SRD analysis were carried out: i) predicted values of the training and test sets together, ii) leave‐one‐out cross‐validated predictions for the training samples.

**Figure 2:**
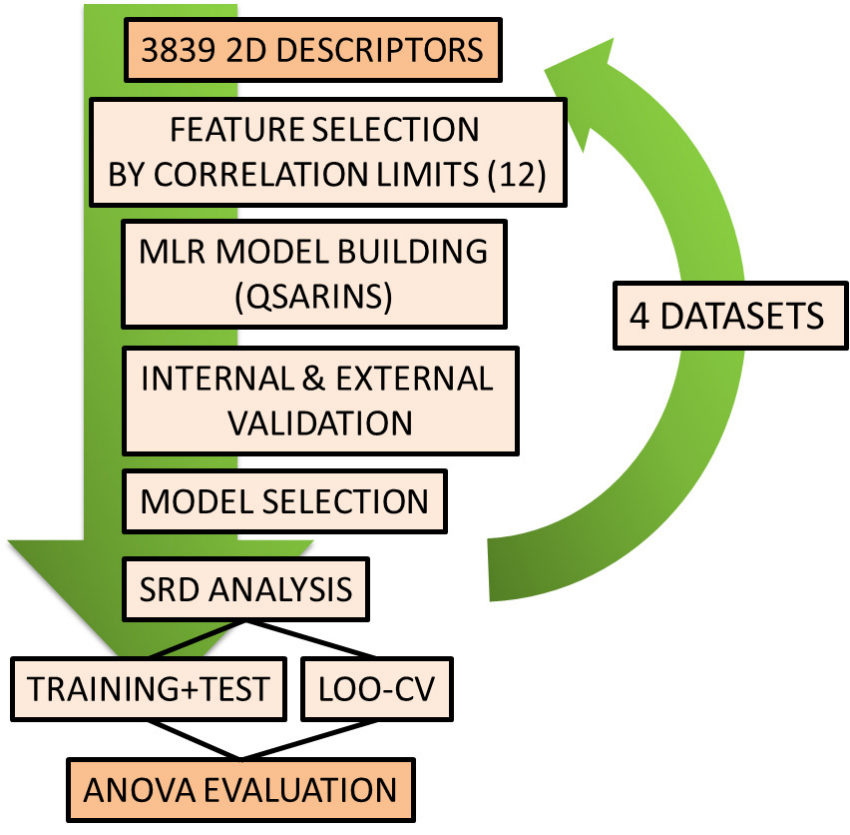
Workflow of the applied procedure from descriptor generation to ANOVA.

Analysis of variance (ANOVA) was used for the statistical comparison of the SRD results for the four datasets. This method is based on the pairwise comparison of the average values of the different groups of samples. Cross‐validated SRD values were used (28 rows for each dataset) for the analysis and the intercorrelation limits were used as groups. STATISTICA 13 (TIBCO Software Inc., Palo Alto, USA) was applied for the ANOVA evaluation. The complete workflow of the procedure is summarized in Figure 2.

## 3 Results and Discussion

**Figure 3:**
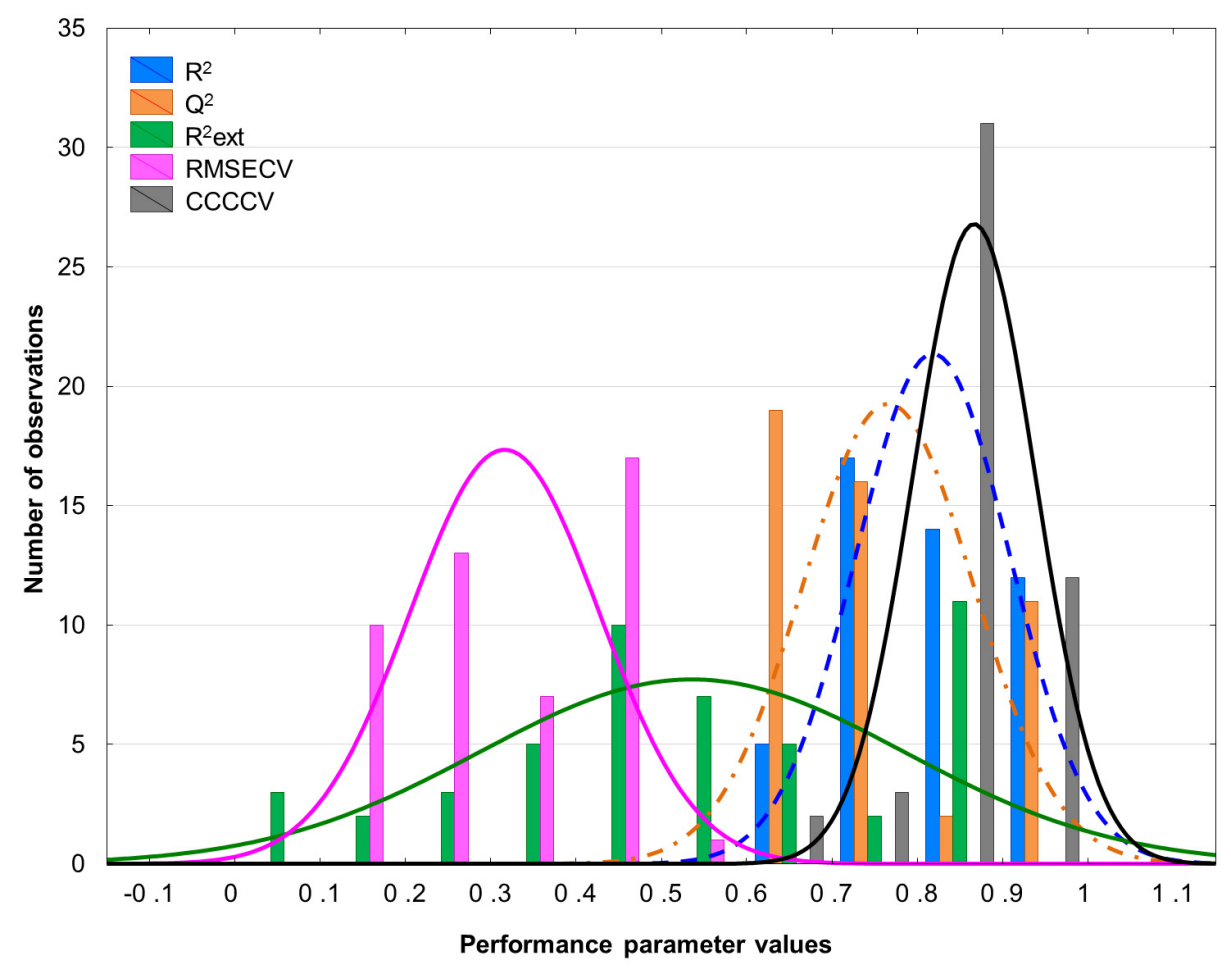
Distribution of the examined performance parameter values. The blue dashed line means the *R* ^2^ distribution, and the orange dashed‐dotted line means the *Q* ^2^ distribution.

**Figure 4:**
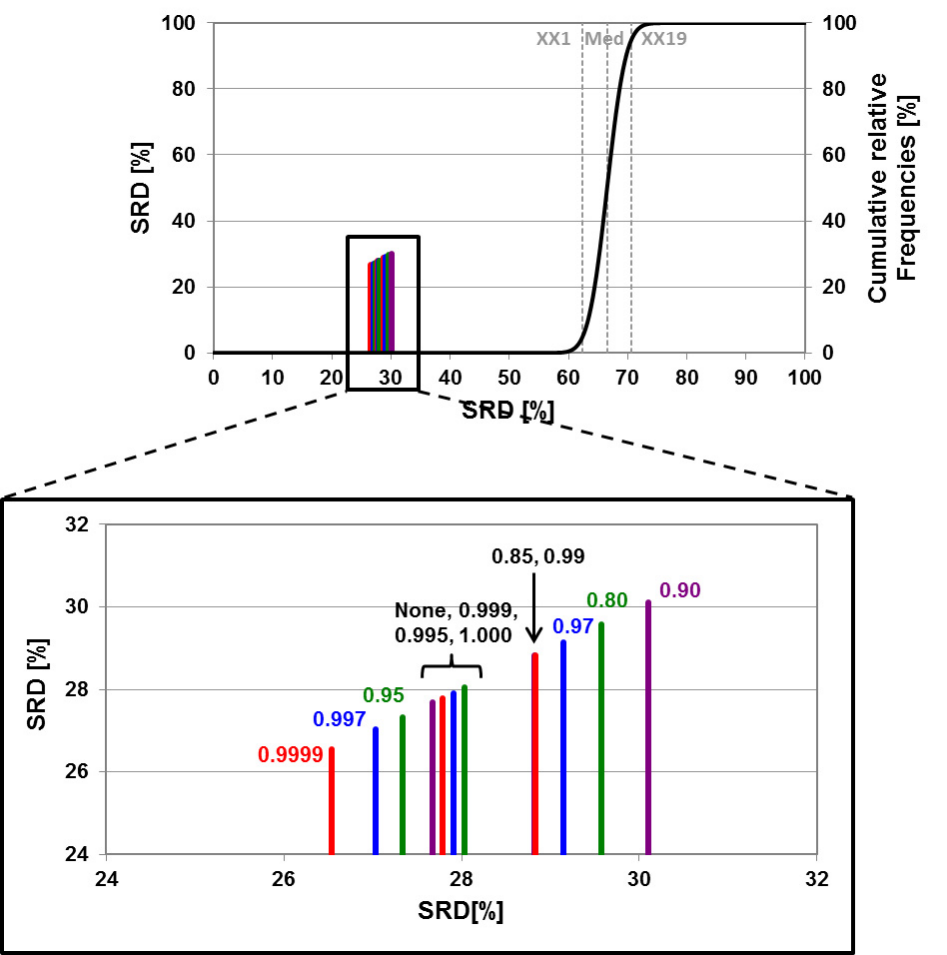
An example SRD result with the full plot (above) and a magnified part (below). Vertical bars denote the models with different intercorrelation limits. The black curve corresponds to the cumulative distribution of SRD values based on random rankings. On the left Y and X axes, normalized SRD [%] values are plotted, while the right Y axis shows the percentages for the distribution of random rankings.

As a first level of comparison, some important performance parameters of the models were compared, to check if these parameters can differentiate between the models with different intercorrelation limits. The goodness of fit for the calibration (*R* ^2^), cross‐validation (*Q* ^2^), and external validation (*R* ^2^_ext_), root mean squared error of cross‐validation (RMSE_CV_) and coefficient of concordance (CCC_CV_)^36^ parameters were selected for this evaluation based on our previous findings.^6^ Figure 3 shows the distribution of these values for the four datasets. It can be clearly seen that the *R* ^2^, *Q* ^2^ and CCC_CV_ values are very close to each other and they have narrow distributions. The (Pearson) correlation coefficient between *R* ^2^ and *Q* ^2^ values for the datasets was higher than 0.90. On the other hand, *R* ^2^_ext_ has a much wider distribution. The RMSE_CV_ values are more dataset dependent, but they are on the same scale. The same information, broken down to separate datasets, is shown in Supplementary Material Figure S1, highlighting the limited discriminatory power of these performance parameters (at least for these datasets). Thus, the classical performance parameters, though well‐known and accepted, are inadequate to unravel small changes and effects. Since SRD was recently shown to surpass them in this capacity,^41^ we have decided to employ it for further analysis. The SRD analysis was based on the predicted endpoints, according to each model, and was carried out for each dataset in two variations: i) with the use of predicted values for the training and test set molecules, and ii) with the use of leave‐one‐out cross‐validated values for the training set molecules (omitting the test set). One example of the SRD results can be seen in Figure 4.

In the case illustrated in Figure 4, all models were much better than the use of random numbers. The vertical bars denote the models with different intercorrelation limits. From the SRD results, we used 28 rows of 7‐fold Monte Carlo cross‐validated SRD values (for each intercorrelation limit) for the comparison with ANOVA. An example of these values can be seen in Supplementary material Table S1 (Dataset 2, leave‐one‐out cross‐validation).

The intercorrelation limit as the categorical factor in the ANOVA procedure was statistically significant based on the results of the four datasets (α=0.05) one‐by‐one and together as well. The outcome of the ANOVA analyses can be found in Supplementary Material Table S2. Thus, the use of different intercorrelation limits during descriptor selection has a significant effect on the final outcome of QSAR model building.

**Figure 5:**
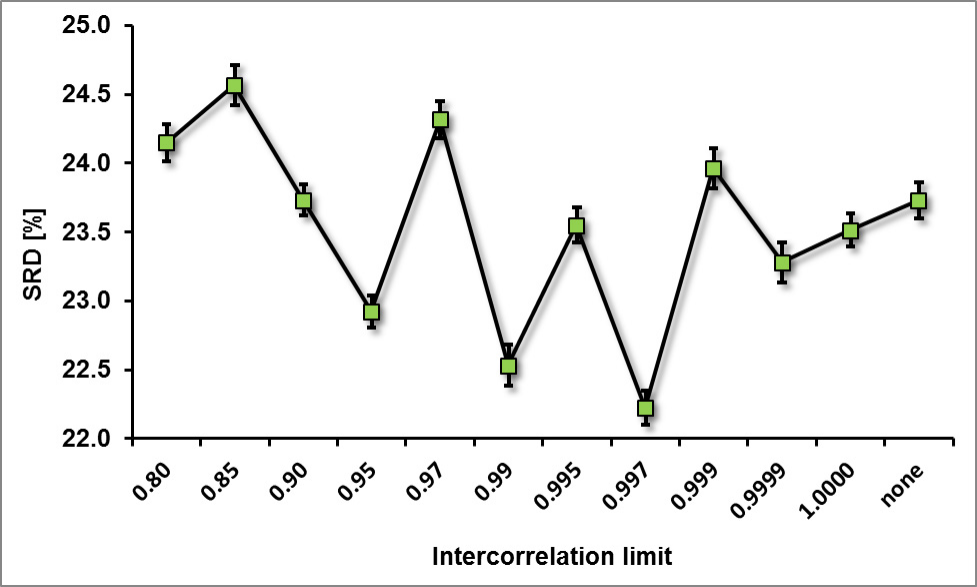
Average normalized SRD values are plotted against the intercorrelation limits. Vertical error bars are calculated based on the standard deviations, with the law of error propagation.

A comparison based on average values across the four datasets, and variances (calculated by error propagation^41^) is shown in Figure 5. This step was necessary because SRD values depend on the performance of the models. (If a dataset provides better models, they will have smaller SRD values in the normalized form as well.) As a consequence, we can compare the error bars only if we take into account the law of error propagation.^42^

As shown in Figure 5 and the separate results for the four datasets (Supplementary Figure S2), the use of an intercorrelation limit for the descriptors is always recommended, but choosing a specific value is not straightforward. In general, a too low value (such as 0.80 or 0.85) usually deteriorates the results. On the other hand, the range between 0.95 and 0.9999 can always yield one or two specific values that present a significant improvement regarding the resulting models.

## 4 Conclusions

Molecular descriptor selection plays an important role in QSAR/QSPR model building. This is usually not reported in detail in research articles, but based on our findings, the choice of intercorrelation limits during molecular descriptor preselection has a significant effect on the outcome. SRD and ANOVA analyses of the applied four datasets show that overall the lower (around 0.80) limits deteriorate the resulting models (by removing valuable descriptors). The region between 0.95 and 0.9999 is applicable (recommended) for variable reduction, keeping in mind that it is worth to check more than one limit before finalizing the selection, as the specific choice is inherently dataset‐dependent. Also, we have shown that even a seemingly insignificant change (like a setting of 0.9999 instead of 0.999) can remove a significant number of descriptors. As such, in addition to proposing the above detailed approach for selecting the intercorrelation limit, we would strongly suggest to authors of future QSAR studies to disclose the specific intercorrelation limit they applied, for the sake of the reproducibility of the whole modeling workflow.

## Conflict of Interest

None declared.

## Supporting information

As a service to our authors and readers, this journal provides supporting information supplied by the authors. Such materials are peer reviewed and may be re‐organized for online delivery, but are not copy‐edited or typeset. Technical support issues arising from supporting information (other than missing files) should be addressed to the authors.

Supplementary
