# Supplementary material for: Intercorrelation Limits in Molecular Descriptor Preselection for QSAR/QSPR
Source: Mol Inform. 2019 Apr 4;38(8-9):1800154. doi: 10.1002/minf.201800154 (PMC6767540; doi:10.1002/minf.201800154)

Supporting Information

© Copyright Wiley-VCH Verlag GmbH & Co. KGaA, 69451 Weinheim, 2019

Intercorrelation Limits in Molecular Descriptor Preselection for QSAR/QSPR

Anita Rácz, Dávid Bajusz, and Károly Héberger*© 2019 The Authors. Published by Wiley-VCH Verlag GmbH & Co. KGaA.
This is an open access article under the terms of the Creative Commons Attribution License, which permits use, distribution and reproduction in any medium, provided the original work is properly cited.
The copyright line for this article was changed on Juli 26, 2019 after original online publication.

Electronic supplementary material:

Intercorrelation limits in molecular descriptor preselection for QSAR/QSPR

Anita Rácz[1], Dávid Bajusz[2], Károly Héberger[1,*]

[1] *Plasma Chemistry Research Group, Research Centre for Natural Sciences, Hungarian Academy of Sciences, H-1117 Budapest, Magyar tudósok krt. 2, Hungary*;

[2] *Medicinal Chemistry Research Group, Research Centre for Natural Sciences, Hungarian Academy of Sciences, H-1117 Budapest, Magyar tudósok krt. 2, Hungary*

Supplementary Figure S1. Performance parameter values for the models based on each intercorrelation limit, for each dataset.

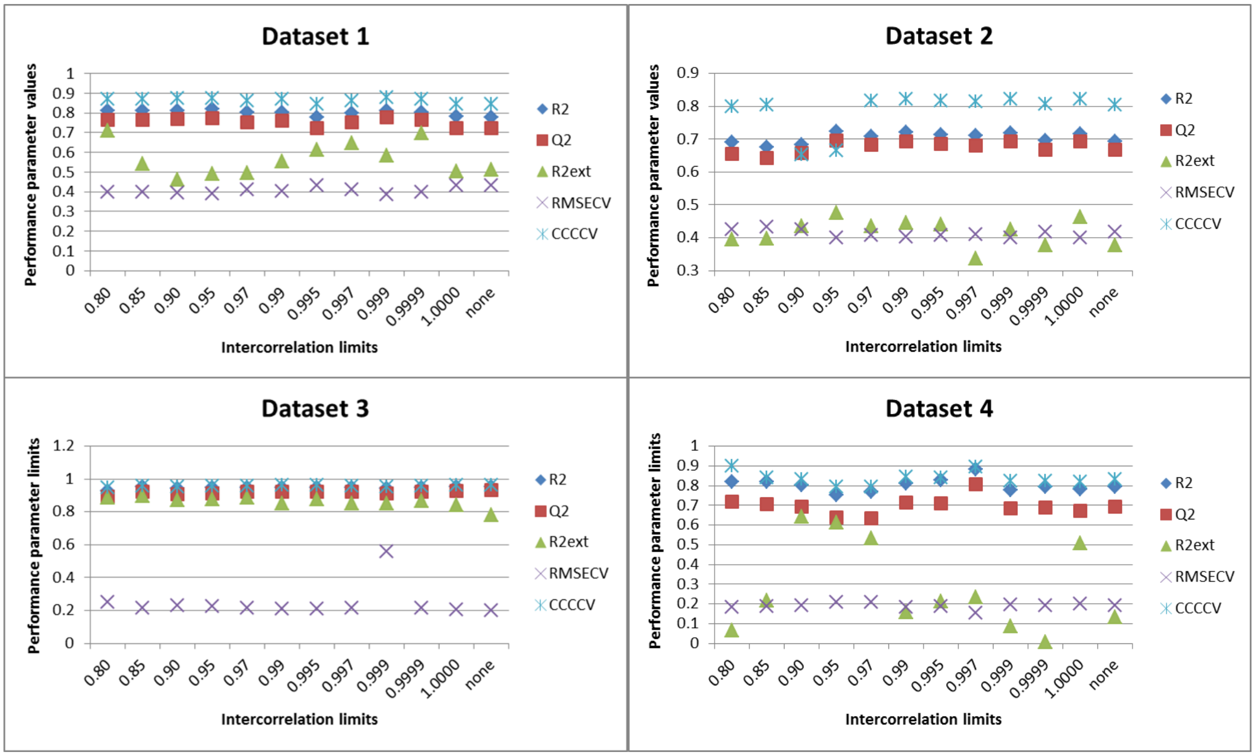

Supplementary Table S1. Example of the validated SRD values for one dataset (Dataset 2 - leave-one-out cross-validation).

	**0.80**	**0.85**	**0.90**	**0.95**	**0.97**	**0.99**	**0.995**	**0.997**	**0.999**	**0.9999**	**1.000**	**none**
**1**	28.7	28.5	29.9	26.7	28.3	28.4	27.5	26.4	27.6	25.9	27.2	26.8
**2**	32.0	30.3	31.5	28.0	29.8	29.9	29.3	28.2	28.5	27.8	29.2	29.4
**3**	28.1	28.6	28.7	26.4	28.3	28.1	26.8	26.0	27.1	25.8	27.6	26.8
**4**	29.5	29.3	30.7	26.7	28.6	28.4	27.3	27.3	27.5	26.4	27.4	27.4
**5**	28.5	27.3	29.1	26.2	28.7	27.4	26.8	26.4	27.2	25.2	27.0	27.3
**6**	31.1	29.9	31.8	29.6	32.1	31.3	29.5	28.4	30.2	28.6	30.1	29.8
**7**	29.3	27.8	29.8	27.9	28.7	28.9	27.8	26.8	28.0	26.5	28.0	26.5
**8**	28.9	27.6	29.0	27.7	28.3	28.1	27.4	26.6	27.3	26.4	27.6	26.5
**9**	32.0	31.1	33.3	30.6	32.7	32.2	30.7	29.4	31.0	29.0	30.9	30.5
**10**	28.6	27.1	28.7	26.3	29.2	27.9	26.8	26.4	27.6	25.3	27.4	27.2
**11**	29.5	29.1	30.4	26.8	28.4	28.0	27.1	27.1	27.3	26.2	27.3	27.3
**12**	28.0	28.3	28.9	26.2	28.2	27.9	26.5	25.9	26.9	25.9	27.3	26.8
**13**	31.0	29.9	30.6	27.4	29.2	29.1	28.5	27.4	27.9	26.7	28.5	28.5
**14**	30.0	29.1	30.8	27.2	28.9	29.1	28.3	27.0	28.2	27.0	27.9	27.6
**15**	29.5	29.0	30.7	27.6	29.6	29.8	28.0	27.2	28.6	26.5	28.4	27.7
**16**	30.5	29.4	30.3	27.8	29.0	28.7	28.1	27.6	27.5	26.5	28.3	28.5
**17**	29.2	27.8	28.9	26.2	27.6	26.9	26.3	26.8	26.9	25.5	26.9	27.0
**18**	29.8	28.9	30.6	26.8	29.6	28.8	28.0	27.3	28.9	26.9	28.0	28.6
**19**	30.1	29.1	30.9	27.9	30.0	29.5	28.9	28.2	28.4	27.1	28.7	28.0
**20**	29.9	29.9	30.6	28.1	28.7	29.6	27.4	26.4	27.5	26.7	28.4	27.0
**21**	28.1	28.1	28.6	26.7	28.8	27.9	27.6	26.2	27.1	26.9	27.4	27.6
**22**	30.1	29.3	31.0	27.5	29.8	29.1	28.1	26.7	28.3	27.3	28.3	27.5
**23**	29.4	28.9	29.9	27.5	29.0	29.4	27.8	27.6	28.2	26.8	27.9	27.8
**24**	30.3	28.8	30.2	26.7	28.9	28.7	26.9	26.5	27.4	26.1	27.8	27.3
**25**	28.6	28.1	29.6	26.4	27.5	27.4	26.2	26.6	26.4	26.0	26.2	26.9
**26**	29.6	29.3	30.1	28.0	29.7	29.3	28.7	28.1	28.9	26.9	28.9	28.4
**27**	29.3	27.6	29.3	25.9	27.6	26.8	27.0	25.0	26.7	25.4	26.9	26.4
**28**	30.1	29.8	31.1	28.9	31.2	30.5	29.0	28.0	29.4	27.7	29.8	28.6

Supplementary Table S2. Result of ANOVA, with the intercorrelation limit (“Limit”) as the categorical factor for the four dataset one-by-one and together (Dataset 1,2,3,4 and Total).

**Dataset 1**	**Univariate Tests of Significance for SRD [%]**				
	SS	Degree of freedom	MS	F	p
Intercept	554332.1	1	554332.1	397999.8	0.0000
**Limit**	470.8	11	42.8	30.7	0.0000
Error	919.2	660	1.4		

**Dataset 2**	**Univariate Tests of Significance for SRD [%]**				
	SS	Degree of freedom	MS	F	p
Intercept	396833.4	1	396833.4	173285.1	0.0000
**Limit**	684.1	11	62.2	27.2	0.0000
Error	1511.4	660	2.3		

**Dataset 3**	**Univariate Tests of Significance for SRD [%]**				
	SS	Degree of freedom	MS	F	p
Intercept	131604.5	1	131604.5	104641.5	0.0000
**Limit**	694.1	11	63.1	50.2	0.0000
Error	830.1	660	1.3		

**Dataset 4**	**Univariate Tests of Significance for SRD [%]**				
	SS	Degree of freedom	MS	F	p
Intercept	494608.0	1	494608.0	66535.39	0.0000
**Limit**	2785.6	11	253.2	34.07	0.0000
Error	4906.3	660	7.4		

**Total**	**Univariate Tests of Significance for SRD [%]**				
	SS	Degree of freedom	MS	F	p
Intercept	1489058	1	1489058	39889.23	0.000000
**Limit**	1227	11	112	2.99	**0.000581**
Error	99895	2676	37		

Supplementary Figure S2: Graphical plot of ANOVA results for the four datasets in order (a,b,c,d) with different intercorrelation limits. Normalized SRD (%) values are plotted on the y axis.

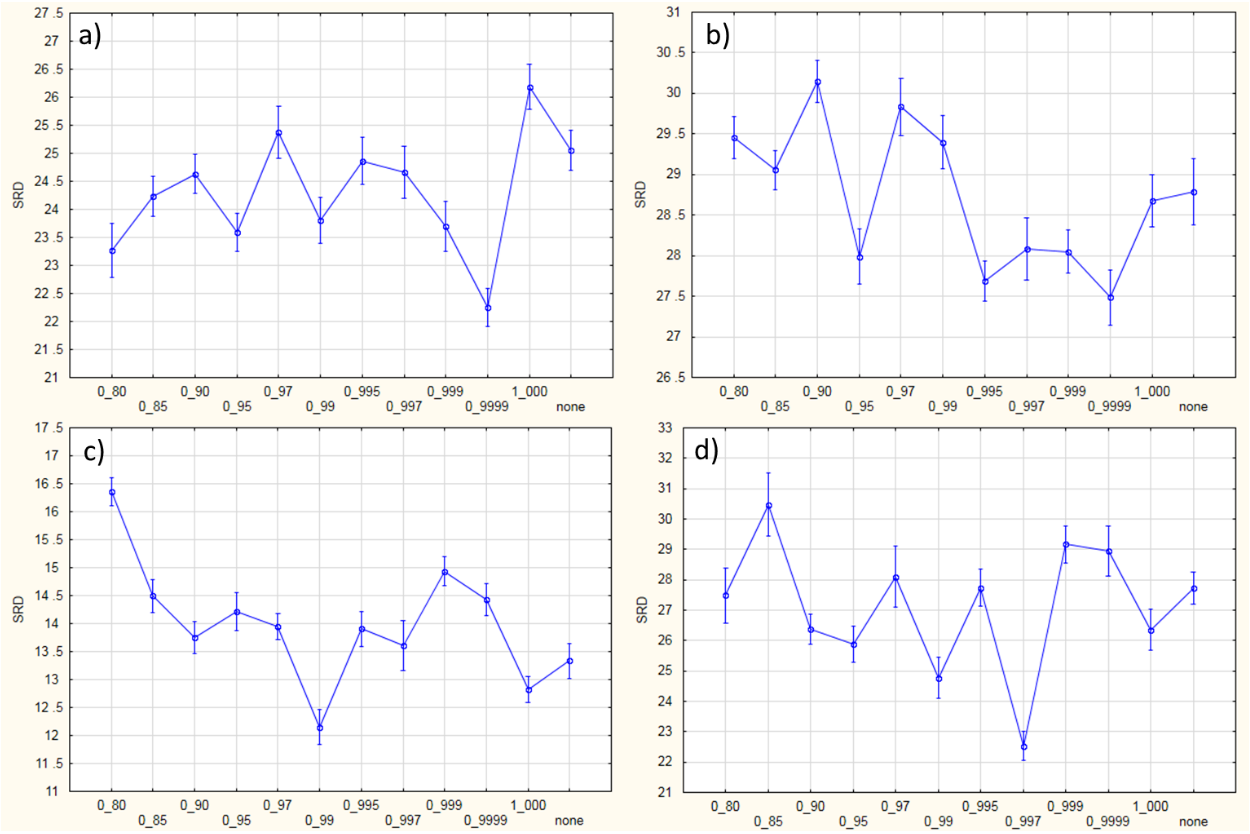

Supplement: Supplementary file 1 — Supplementary [file MINF-38-na-s001.pdf]
